# DAILY ASSOCIATIONS OF SOCIAL INTERACTIONS WITH OBJECTIVE SLEEP MEASURES AMONG OLDER ADULTS

**DOI:** 10.1093/geroni/igad104.0181

**Published:** 2023-12-21

**Authors:** Jee eun Kang, Linying Ji, Orfeu Buxton, Martin Sliwinski

**Affiliations:** Pennsylvania State University, State College, Pennsylvania, United States; Montana State University, Bozeman, Montana, United States; Pennsylvania State University, State College, Pennsylvania, United States; Pennsylvania State University, State College, Pennsylvania, United States

## Abstract

Disturbed sleep is a common problem and can have profound effects on older adults. Social relationships are known to be associated with quantity and quality of sleep. To extend prior research, we examined the associations of daily social interactions and sleep, using objective sleep measures and an ecological momentary assessments (EMA) approach. Participants in the Einstein Aging Study (n=285, Mage=77, range=70-90, 68% women, 46% White, 40% Black) reported their recent social interactions and physical contact four times a day for 16 days via EMA on mobile phones. Objective sleep measures (sleep timing, night sleep time, wake after sleep onset (WASO), sleep maintenance efficiency) were calculated from concurrent wrist actigraphy. Multilevel models controlled for demographics, living arrangement, Instrumental Activities of Daily Living, sleep-disordered breathing, and hypoxemia. Results showed that, on days when older adults had more day-time social interactions than their average, they had earlier sleep timing, shorter night sleep time, and less WASO, but no differences in sleep efficiency. On days when older adults had more physical contact than their average, they had earlier sleep timing and shorter night sleep time but no differences in WASO or sleep efficiency. These micro-longitudinal analyses among older adults suggest that daily social interactions are related to subsequent earlier sleep timing and shorter sleep at night, and can be beneficial for sleep quality. Physical contact seemed to be only associated with the timing and quantity of sleep but not sleep quality. Potential benefits of using an EMA approach in sleep research will be discussed.

